# Mycosis Fungoides Is a Proliferation of Multiple Mutational Subclones

**DOI:** 10.1111/1346-8138.70159

**Published:** 2026-01-27

**Authors:** Ryoma Honda, Toshihisa Hamada, Makoto Sugaya

**Affiliations:** ^1^ Department of Dermatology International University of Health and Welfare Narita Japan; ^2^ Department of Dermatology Kyushu University Fukuoka Japan

**Keywords:** CD30^+^ lymphoproliferative disorders, intratumoral heterogeneity, mycosis fungoides, resident memory T cells

## Abstract

Mycosis fungoides (MF) is a unique type of lymphoma with an excellent prognosis presenting with a wide variety of clinical and histological findings. Recent sophisticated research techniques have revealed that very slow changes in the clinical and histological pictures of one patient over time are due to intratumoral heterogeneity. This means the distribution of different subclones into individual skin lesions and the accumulation of genomic and epigenomic changes in known pathways, starting from immature T‐cell precursors. MF is now recognized as the proliferation of multiple mutational subclones that originate from immature T‐cell precursors with malignant potential. This may explain why there are so many case reports of MF accompanied by T‐cell lymphoproliferative disorders or cutaneous T‐cell lymphomas. In addition, the distinction between “transformation” and “complication” of MF may depend on where the disease branches in the T‐cell lineage development and the extent of the disease entity defined by MF.

## 1 Introduction

Mycosis fungoides (MF) is the most common form of cutaneous T‐cell lymphoma (CTCL) characterized by the proliferation of cerebriform skin‐homing CD4^+^T cells [1]. Although the disease usually has an indolent clinical course, advanced MF is very difficult to treat. Approximately 70% of patients are diagnosed at an early stage (stages IA to IIA), with only a limited number of cases progressing to an advanced stage [2, 3]. We should avoid using multi‐drug chemotherapy for early stage MF cases [4]. On the contrary, we should take a long‐term view when following up patients with MF, just as we do with those with phacomatosis. Although phacomatosis is a congenital disease with common hotspot mutations and MF is a non‐congenital malignant disease, the two conditions exhibit some clinical similarities. The clinical and histological manifestations of MF can vary widely from patient to patient, which is characteristic of this type of lymphoma. Even one patient can have patches and tumors as well as small/medium‐sized tumor cells and large anaplastic cells. The very slow changes in the clinical and histological features of one patient over time are also unique to this lymphoma. The morphological and phenotypic diversity of MF cells, both between and within lesions, presents challenges in terms of diagnosis and treatment. Recent advances in the research in this field have clearly revealed the background of “transforming” nature of this disease. In this review, the pathogenesis of this unique lymphoma and the idea of handling the disease are described.

## 2 How Do Tumor Cells Travel to Other Skin Sites in MF?

MF accounts for about a half of cutaneous lymphoma, a finding that was consistent across Japan, the United States, and Europe. Lymphomas of T/NK‐cell origin account for about 70%–80% of cutaneous lymphomas [3, 5, 6]. In contrast, nodal lymphoma is characterized by the predominance of B‐cell lymphomas. The reason for this organ specificity can be simply explained by the physiological roles of T and B cells. Some T cells populate peripheral barrier tissues such as skin, intestines, and liver, where they develop into organ‐specific resident memory T cells (T_RM_) and do not circulate [7]. T_RM_ are essential for the primary immune response and express several important surface markers, including CD69 and CD103 [8, 9]. B cells, on the other hand, are mainly in the lymphoid tissues such as lymph nodes and spleen and produce antibodies to fight invaders. Therefore, it is very rare to find B cells in skin biopsies, not only in cases of cutaneous lymphoma but also in inflammatory skin diseases such as psoriasis and atopic dermatitis. It is widely accepted that lymphoma cells develop from their normal counterparts in tissue. It is therefore natural that T‐cell lymphomas are dominant in the skin.

**FIGURE 1:**
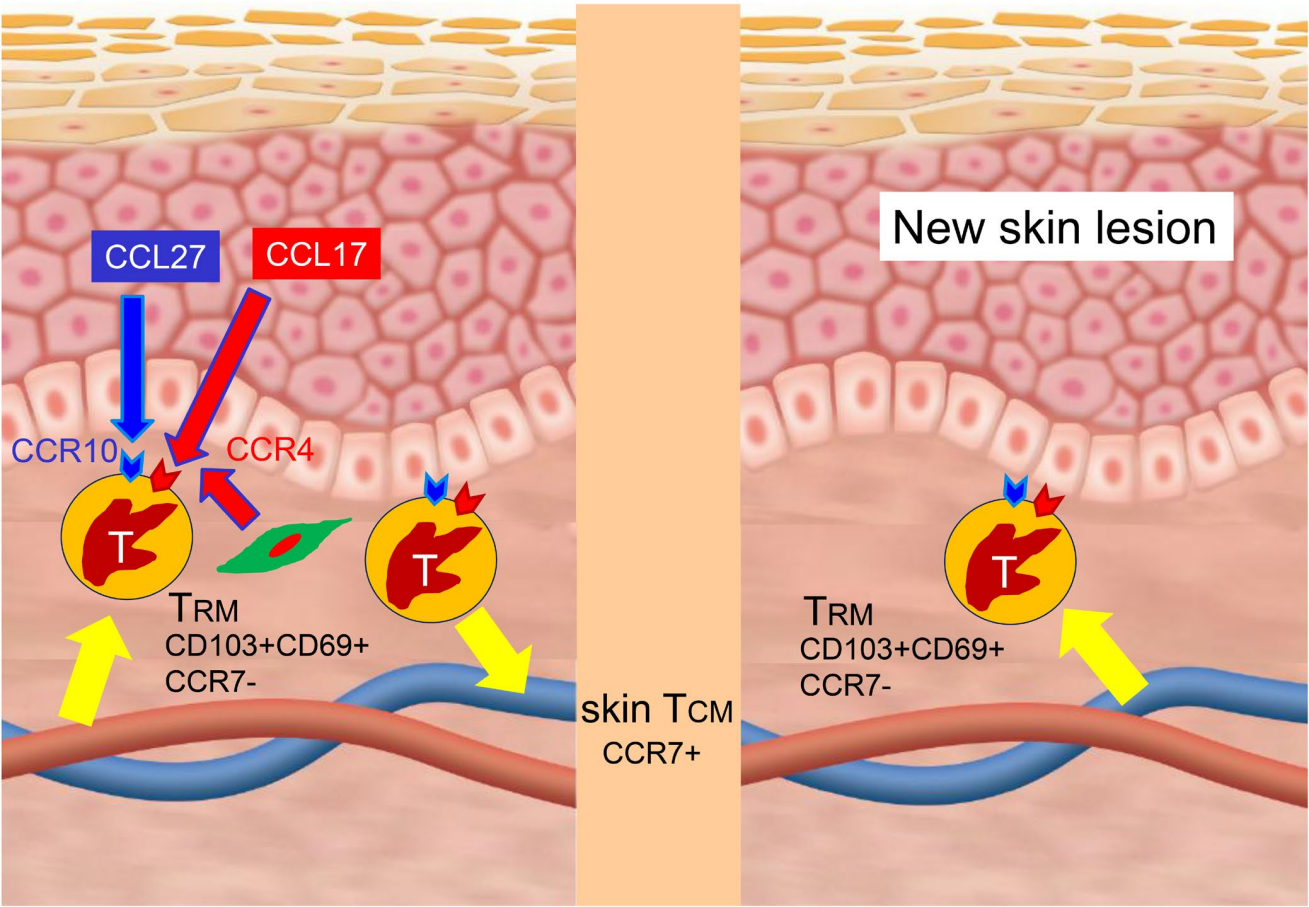
The mechanism of lesional dissemination in mycosis fungoides: T_RM_ and T_CM_ dynamics. Tumor cells in mycosis fungoides (MF) are believed to originate from skin‐resident memory T cells (T_RM_). While T_RM_ were traditionally thought to be tissue‐anchored, recent evidence suggests that they can leave the skin and convert into circulating central memory T cells (T_CM_), known as “ex‐T_RM_.” These T_CM_ may return to the skin, giving rise to new lesions. This plasticity could explain the multifocal lesion development in MF despite minimal peripheral blood involvement.

Chemokines play a pivotal role in guiding T cells to specific tissues. Many resident cells in the skin, including Langerhans cells and fibroblasts, express CCL17, which is important for the migration of malignant T cells that express CCR4 [10, 11, 12, 13, 14, 15, 16, 17]. Mogamulizumab, an antibody against CCR4, has been useful in treating advanced stage MF [18]. It is widely accepted that MF is a malignant proliferation of T_RM_ which resides in the skin for a long time. SS originates from central memory T cells (T_CM_), which can travel all over the body via the bloodstream [19, 20, 21, 22, 23]. T_CM_ express high levels of CD62L and CCR7, enabling them to maintain their circulatory capacity [24]. This theory of two distinct types of CTCL well explains the presence of well‐demarcated skin lesions and the absence of blood involvement in MF. Erythroderma and blood and lymph node involvement are also reasonable if the tumor cells in SS are T_CM_. MF patients, however, usually have multiple skin lesions even in the early stages. New skin lesions can develop during the course of the disease. It has been a big question how tumor cells travel to other skin sites in MF, since tumor cells are not typically detected in the peripheral blood of most MF patients using traditional methods, such as flow cytometry or T‐cell receptor (TCR) gene rearrangement analysis via Southern blotting. Recent, sophisticated studies have revealed several clones in the blood and skin of patients with MF [25, 26, 27]. A malignant skin clone can be detected in the blood, even when it is not the most dominant blood clone [28, 29]. Furthermore, plasticity between T_CM_ and T_RM_ has been reported, demonstrating that skin T_RM_ exhibit novel circulating characteristics in both mice and humans. Several studies have indicated that T_RM_ can egress from the tissue into the blood as so‐called “ex‐T_RM_”, which retain the biased homing and differentiation potential [30, 31]. Matsumura et al. reported that T_CM_ was abundantly found in recurrent lesions of fixed drug eruption developed from lesional T_RM_ rather than blood T_CM_ [32]. In some cases, generalized fixed drug eruption can clinically resemble MF [33]. Since tumor cells in MF originate from skin‐resident T_RM_, the plasticity between T_CM_ and T_RM_ may explain how malignant T cells in MF travel to other areas of the skin and cause lesions (Figure 1).

## 3 Intratumoral Heterogeneity in MF

**FIGURE 2:**
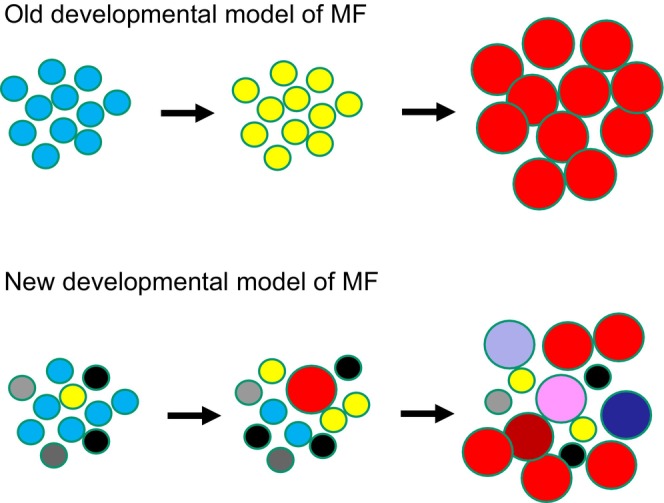
Intratumoral heterogeneity and subclonal architecture in skin lesions of mycosis fungoides. Single lesions in mycosis fungoides (MF) often contain multiple tumor subclones, each with distinct somatic mutations. This subclonal complexity increases with disease progression. Clonal expansion and divergence across lesions may account for the morphological and histological variability observed within individual patients and contribute to immune evasion and resistance to therapy.

Intratumoral heterogeneity (ITH) is a widely accepted concept in various malignancies. It refers to the distribution of somatic mutations among subsets of malignant cells (subclones) rather than in all malignant cells (clonal mutations) [34, 35, 36]. Recent studies have shown that MF is a highly heterogeneous neoplasm composed of multiple mutational subclones rather than a single aggressive clone that progresses in a linear fashion (Figure 2) [37, 38, 39, 40]. Even in the early stages, tumor cells in MF show vast clonotypic diversity within single skin lesions and between different lesions [41]. As the disease progresses, ITH increases, which can explain how tumor cells escape immune surveillance and why each skin lesion exhibits unique clinical and histological characteristics in the same patient. ITH can also explain how tumor cells become resistant to anti‐cancer drugs [42]. Pseudotime analysis revealed the seeding phenomenon of tumor cells from tumorous skin lesions to surrounding skin [25, 43]. This suggests that tumorous lesions can provide malignant subclones.

## 4 Where Do Tumor Cells Originate in MF?

Specific chromosomal translocations are known to be critical for the pathogenesis of some lymphomas. For instance, *ALK* translocation is essential for developing a subset of systemic anaplastic large cell lymphoma (ALCL) [44, 45]. Translocation of *DUSP22* has been found in 20%–57% of primary cutaneous ALCL (pcALCL) [46, 47, 48, 49]. Conversely, no disease‐specific driver mutations have been discovered in MF [50]. However, previous analyses of the genomic landscape have revealed over 50 driver mutations in known pathways, such as NF‐κB, NOTCH, and JAK–STAT signaling, as well as disturbances in biochemical mechanisms involving DNA repair and chromatin stability [51, 52, 53, 54, 55, 56, 57, 58, 59]. Based on new evidence of significant heterogeneity in the TCR repertoire and genomic changes of MF tumor cells, it is believed that malignant transformation occurs during early T‐cell development. Various clones with common genomic mutations spread to the skin and only clones with additional genetic and epigenetic advantages survive and cause skin lesions. In fact, a case was reported in which MF tumor cells had a *TBL1XR1/TP63* fusion, which was detected in a resected lymph node before the onset of MF [60]. The frequency of monoclonal TCRγ rearrangements was similar to that of tumor cells in some MF skin biopsy samples, while multiple TCRγ, TCRα, and TCRβ clonotypes were detected by whole exome sequencing analysis in most MF samples. This finding supports the theory that tumor cells in MF originate from lymphoid precursors, rather than mature T cells in peripheral organs such as skin, before TCRβ or TCRα rearrangements [61]. This theory explains why T‐cell lymphoproliferative disorders are frequently seen in MF patients. Further research is needed to determine whether common lymphoid progenitors or other mechanisms are responsible for B‐cell lineage malignancies, including Hodgkin lymphoma, which are more prevalent in MF patients than in healthy individuals [62, 63, 64, 65].

## 5 CD30 ^+^ Lymphoproliferative Disorders in MF Patients

**FIGURE 3:**
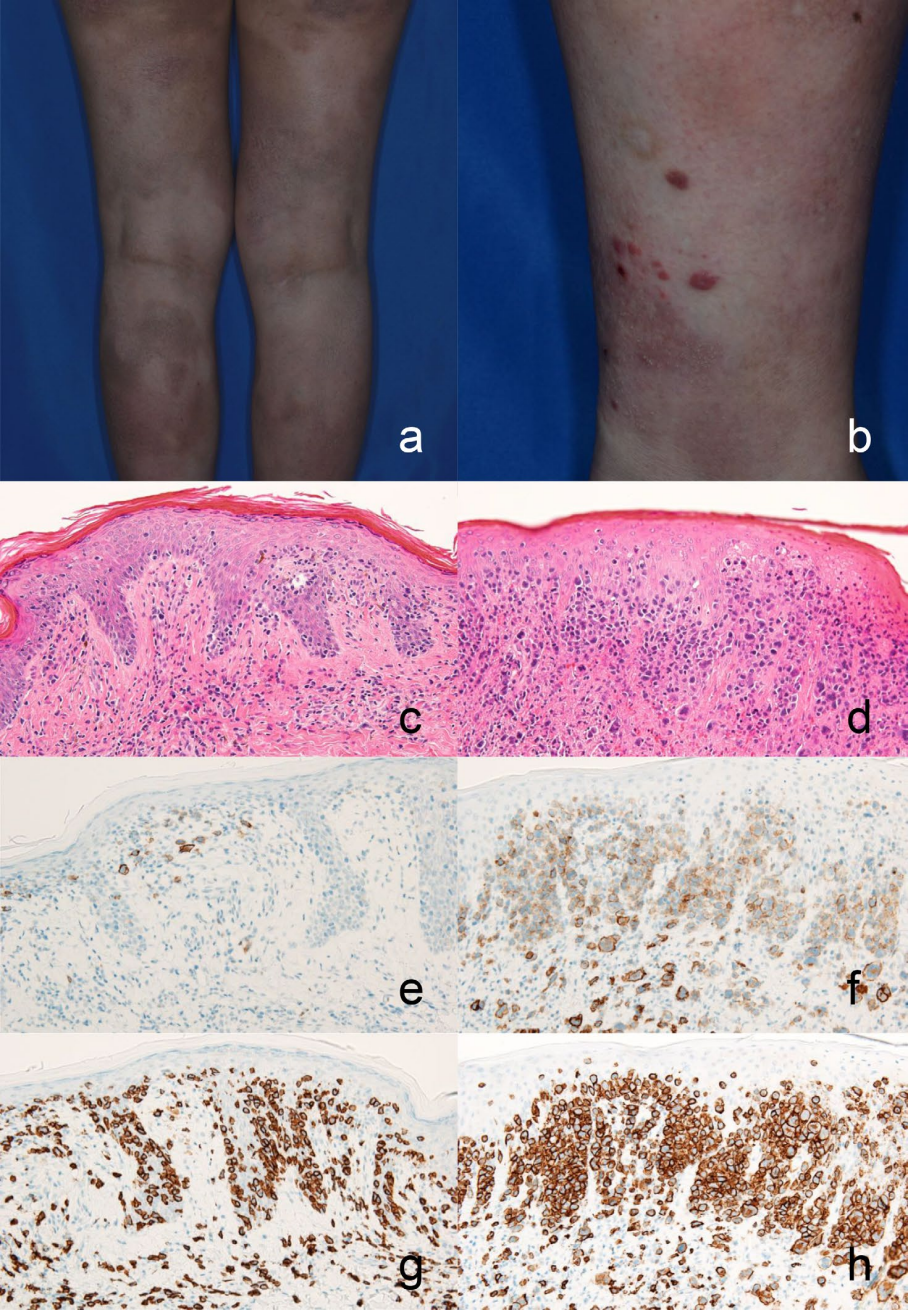
A case with mycosis fungoides associated with lymphomatoid papulosis type D. (a, b) Clinical picture of mycosis fungoides (MF) associated with lymphomatoid papulosis (LyP). Scaly patches (a) and reddish papules (b) on the lower extremities. (c, d) H&E staining of biopsy specimens. Epidermotropism of tumor cells is seen in both MF patch (c) and LyP (d), while the latter shows many large atypical cells infiltrating. (e, f) Staining for CD30 in MF patch (e) and LyP (f). (g, h) Staining for CD8 in MF patch (g) and LyP (h).

If CD30^+^ large T cells are detected during the course of MF, large cell transformation (LCT) or a complication of lymphomatoid papulosis (LyP) is a possible diagnosis. The combination of MF and pcALCL is not currently used as a diagnostic term because it is impossible to distinguish from LCT. However, previous studies clearly showed that the tumor cells of MF and those of accompanied LyP were from the same clone using traditional analysis. This made it impossible to identify pathological differences between the complication of LyP and LCT in MF [66, 67, 68, 69]. There are six subtypes of LyP. The most common subtype is LyP type A, which is characterized by the infiltration of large anaplastic CD30^+^ cells in a mixed inflammatory background that includes neutrophils, eosinophils, and small lymphocytes. LyP type B is defined by a band‐like infiltration of small‐ to medium‐sized cerebriform lymphocytes, which is histologically indistinguishable from MF. CD30 is sometimes negative. Type C LyP is characterized by sheets of large anaplastic CD30^+^ lymphocytes with fewer admixed inflammatory cells. Without clinical information, it is almost impossible to differentiate this type of LyP from pcALCL. Type D LyP shows prominent epidermotropism of atypical, small‐ to medium‐sized CD8^+^ and CD30^+^ pleomorphic lymphocytes. LyP type E is characterized by angioinvasive and angiodestructive features, and it can present with necrotic or ulcerative lesions. The sixth type of LyP is identified by *DUSP22* rearrangement and exhibits biphasic morphology with two distinct cellular components. Many clinicians believe that the CD30^+^ large cells in type A LyP are tumor clones and CD30^−^ small cells are reactive T cells. However, Gellrich et al. reported that the CD30^−^ small cells in type A LyP are monoclonal. We speculate that both the CD30^+^ large cells and some of the CD30^−^ small cells in LyP type A are subclones that originate from the same lymphoid precursors as in MF [70], although further evidence is necessary. The clinicopathological overlap between papular MF and LyP type B, in which tumor cells are negative for CD30, may represent a continuous spectrum of the two diseases [71, 72, 73]. We also observed an MF case associated with LyP type D, in which the histological findings of the MF patch lesion and LyP were similar except for the frequency of CD30^+^ large cells (Figure 3). Taken together, we speculate that certain types of LyP, such as types A, B, and D, might be pathologically closer to MF than to pcALCL, although further study is needed.

**FIGURE 4:**
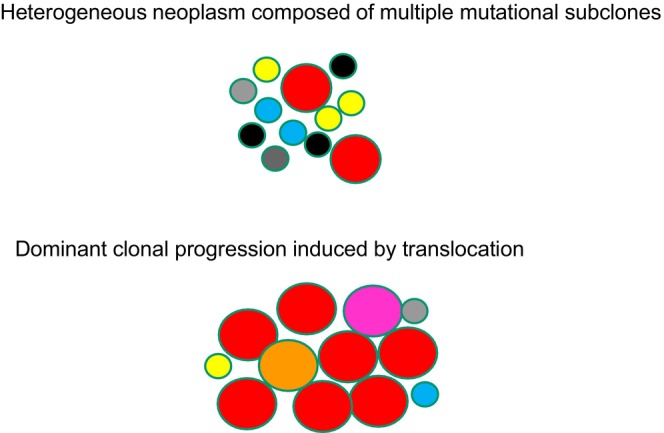
A heterogeneous neoplasm composed of multiple subclones versus dominant clonal progression induced by a translocation. Some hematological malignancies such as mycosis fungoides and subtypes of lymphomatoid papulosis have overlapping clonal origins and exhibit intratumoral heterogeneity, suggesting a branched clonal evolution. In contrast, other neoplasms such as translocation‐positive primary cutaneous anaplastic large cell lymphoma arise through a single oncogenic translocation, such as *ALK* or *DUSP22* rearrangement, indicating linear progression of dominant clones.

Various types of genomic translocations have been identified in systemic ALCL. Translocation partners of *ALK*, other than *NPM*, have been reported [74, 75, 76]. In pcALCL, rearrangement of the *DUSP22‐IRF4* locus on 6p25.3 has been identified in 20%–57% of cases [45, 46, 47, 48]. Other than *DUSP22* rearrangement, *TP63* rearrangement has been reported in approximately 10% of ALK‐negative systemic ALCL and pcALCL cases [77]. Since translocations are more prevalent in pcALCL than previously thought, we hypothesize that CTCLs can be classified into two categories: (1) a heterogeneous neoplasm comprising multiple mutational subclones, such as MF or subtypes of LyP, and (2) dominant clonal progression driven by translocations, including *DUSP22*‐rearranged LyP or translocation‐positive pcALCL (Figure 4). Although clonotypic diversities have been reported in the latter group, targeting the chimeric protein remains a promising therapeutic strategy, as the ALK inhibitor Alectinib is an effective treatment for relapsed or refractory ALK‐positive ALCL.

## 6 Difficulty in Definition of “Transformation” or “Complication” of MF

**FIGURE 5:**
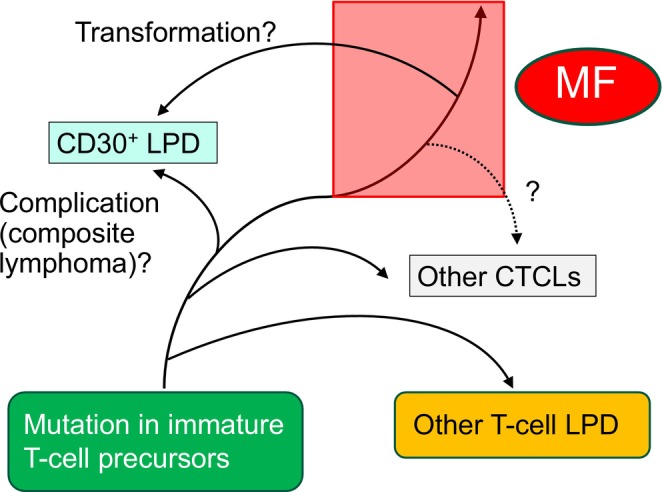
Mycosis fungoides is a neoplasm that develops from immature T‐cell precursors with malignant potential. Mycosis fungoides (MF) is a neoplasm that develops from immature T‐cell precursors with malignant potential, which is also true for other cutaneous T‐cell lymphomas. The difference between “transformation” and “complication” of MF may depend on where the disease branches and how large an area the diagnosis of MF should cover.

The wide variety of clinical and histological presentations and their gradual changes over time are specific to MF among CTCLs. The distribution of varieties of subclones into each skin lesion and the accumulation of genomic and epigenomic changes in known pathways, beginning with immature T‐cell precursors, may explain MF's “transforming” nature. We have encountered numerous cases of systemic ALCL accompanied by the patch stage of MF. Phenotypic changes in tumor cells, including a shift from CD4^+^ to CD8^+^, have been reported in some cases [78, 79]. Additionally, cases of aggressive epidermotropic cytotoxic T‐cell lymphoma following long‐term MF‐like skin eruptions have been reported [80]. We also reported a case of γδ T‐cell lymphoma that developed from MF [81]. If MF arises from skin T_RM_ that differentiates from immature T‐cell precursors with malignant potential, it is not surprising that there are many case reports of accompanying T‐cell lymphoproliferative disorders or other CTCLs with MF (Figure 5). Indeed, recent studies have revealed that other CTCLs are clonotypically oligoclonal as well, suggesting that they originate at the level of lymphoid precursors [82, 83]. Various opinions have been expressed regarding the “transformation” versus “complication (composite lymphoma)” of MF, particularly with regard to CD30^+^ lymphoproliferative disorders (LPDs). If the diagnosis of CD30^+^ LPDs is made before that of MF, it can be called a composite lymphoma of MF and CD30^+^ LPDs. If both lymphomas are diagnosed simultaneously or if CD30^+^ LPDs develop after the diagnosis of MF, the transformation of MF tumor cells cannot be ruled out. However, the difference between “transformation” and “complication” is not so clear in Figure 5. It depends on where the branching of the disease occurs in the T‐cell lineage development and how large the area defined by the disease entity should be (the red area in Figure 5). Regarding the differences between MF and parapsoriasis en plaque (PP), most, but not all, small plaque lesions were reported to be polyclonal and characterized by *NPY* ^+^ *CRISP3* ^+^ innate lymphoid cells. Conversely, three‐fourths of large plaque patients showed expansion of a single, dominant TCR clone [84]. Therefore, most large plaque‐type PP lesions may also be included in the red area in Figure 5. Furthermore, we speculate that some CTCLs result from the transformation of MF tumor cells, given that MF is a highly heterogeneous neoplasm composed of multiple mutational subclones (dotted line in Figure 5). This idea could resolve the debate over the diagnosis of controversial cases, such as rare types of CTCL, MF, or complications of the two diseases, although further reports including clonal analyses are required.

## 7 Conclusion

Recent advances in cutaneous disease research, made possible by sophisticated techniques such as single‐cell RNA sequencing, whole genome sequencing, and TCR repertoire analysis, have changed our understanding of their pathogenesis. MF is now recognized as the proliferation of multiple mutational subclones rather than a single aggressive clone. If MF arises from skin T_RM_ that differentiates from immature T‐cell precursors with malignant potential, the numerous case reports of accompanying T‐cell lymphoproliferative disorders or other CTCLs with MF are not surprising. Additionally, the distinction between “transformation” and “complication” of MF may depend on where the disease branches in the T‐cell lineage development and how large the disease entity defined by MF should be.

## Funding

The authors have nothing to report.

## Conflicts of Interest

The authors declare no conflicts of interest.

## Data Availability

Data sharing not applicable to this article as no datasets were generated or analyzed during the current study.
